# Role of *GNB3*, *NET*, *KCNJ11*, *TCF7L2* and *GRL* genes single nucleotide polymorphism in the risk prediction of type 2 diabetes mellitus

**DOI:** 10.1007/s13205-016-0572-x

**Published:** 2016-12-02

**Authors:** Saliha Rizvi, Syed Tasleem Raza, Qamar Rahman, Farzana Mahdi

**Affiliations:** 1 Department of Biochemistry, Era’s Lucknow Medical College and Hospital, Lucknow, 226003 India; 2 Amity University, Lucknow Campus, Lucknow, Uttar Pradesh India

**Keywords:** Genetic polymorphism, *GNB3*, *GRL*, *NET*, *KCNJ11*, *TCF7L2*, Type 2 diabetes mellitus

## Abstract

Type 2 diabetes (T2DM) is a polygenic metabolic disorder characterized by hyperglycemia occurring as a result of impaired insulin secretion or insulin resistance. Various environmental and genetic factors interact and increase the risk of T2DM and its complications. Among the various genetic factors associated with T2DM, single nucleotide polymorphism in different candidate genes have been studied intensively and the resulting genetic variants have been found to have either positive or negative association with T2DM thereby increasing or decreasing the risk of T2DM, respectively. In this review, we will focus on *Guanine nucleotide*-*binding protein subunit beta 3 (GNB3), Norepinephrine Transporter (NET), Potassium Channel gene (KCNJ11), Transcription Factor 7*-*Like 2 (TCF7L2) and Glucocorticoid receptor (GRL)* genes and their association with T2DM studied in different ethnic groups. The products of these genes are involved in the biochemical pathway leading to T2DM. Polymorphisms in these genes have been intensively studied in individuals of different ethnic origins. Results show that genetic variants of *TCF7L2* and *KCNJ11* genes have potential to emerge as a risk biomarker for T2DM whereas results of *GNB3*, *GRL* and *NET* genes have been controversial when studied in individuals of different ethnicities. We have tried to summarize the results generated globally in context to the selected genes which could possibly help researchers working in this field and would eventually help in understanding the mechanistic pathways of T2DM leading early diagnosis and prevention.

## Introduction

Diabetes mellitus is a widely recognized major health issues affecting more than 170 million individuals worldwide. Among the different types of diabetes i.e., type 1 diabetes, type 2 diabetes and gestational diabetes, majority of diabetes cases (~90%) are of type 2 diabetes mellitus (T2DM). Increase in incidences of T2DM is becoming a major concern in developing countries and has emerged as an epidemic worldwide. It is predicted that by 2030 India will harbour 79.4 diabetics followed by China (42.3 million) and the United States (30.3 million) (Kaveeshwar and Cornwall 2014). It is characterized by hyperglycemia occurring as a result of impaired insulin secretion, insulin resistance and increased glucose output by liver or a combination of them (American Diabetes Association 2004). Long standing T2DM increases the risk of associated micro and macro vascular complications such as stroke, dyslipidemia, retinopathy, heart disease, stroke, peripheral neuropathy, nephropathy (Rizvi et al. 2014), blindness and amputation (Brownlee 2001). Various factors and their combined effects have been found to contribute to the development of T2DM such as environmental factors, obesity, lifestyle, family history, drugs, etc. Apart from these factors T2DM have a strong genetic component since it’s a polygenic disease with multiple genes interacting with one another along with other factors especially with various environmental factors contributing to disease susceptibility (Jenkins et al. 2013).

Over the past few decades variants in various candidate genes for T2DM such as *KCNJ11* (Gloyn et al. 2003), *TCF7L2* (Grant et al. 2006), *PPAR*-*G* (Altshuler et al. 2000)*, CAPN10* (Tsuchiya et al. 2006), *GSTT1/M1* (Raza et al. 2014), *FTO* (Raza et al. 2014), *PTPNI* (Florez et al. 2005), etc. have been extensively studied and investigated through genetic linkage analysis and association studies to find their possible association with T2DM and declare them as susceptible genes. Most of the susceptibility genes were found to perform key regulatory functions in various pathways leading to glucose tolerance thus resulting in disease outcome (Lorenzo et al. 2013).

Identification of susceptibility genes is an important area of diabetes research since it help in finding genetic biomarkers which could help in early identification, treatment, and thus prevention of disease in susceptible individuals. Early identification and subsequent treatment strategies would also prevent the risk of developing the risk of secondary complication associated with T2DM in the susceptible individuals. However, these genetic biomarkers interact with various environmental and lifestyle factors to predispose an individual to T2DM risk. Since these environmental and lifestyle factors change with each ethnic group, a gene which shows association with T2DM in one ethnic group might show weak or no association in a different ethnic group. Thus, it is important to replicate the association studies and identify as well as characterize the gene variants related to T2DM in each ethnic group (Yamada et al. 2006).

## Candidate and susceptibility genes for T2DM

The candidate gene approach in T2DM research helps in the identification of those genetic variants which are associated with disease outcome. Meta-analysis studies conducted worldwide on various candidate genes help in the determination of susceptibility genes for a particular disease. However, in case of a polygenic disease the results may not be conclusive since gene–gene interactions also play an important role since the contribution of a gene variant is more profound for a disease phenotype when it is the result of gene–gene interactions. A large number of candidate genes have been studied in the last two decades and enormous data is available from various association studies conducted on a global scale. In this review, we will focus on *GNB3*, *NET*, *KCNJ11*, *TCF7L2* and *GRL* genes and their association with T2DM studied in different ethnic groups as shown in Table 1. The products of these genes are involved in the biochemical pathway leading to T2DM. Products of *GNB3* gene regulate blood glucose levels through metabolic pathway of insulin signaling, while that of *NET* gene is involved in sodium–chloride dependent active reuptake of norepinephrine which in turn increases release of glucagon from pancreas thereby increasing blood glucose levels. *KCNJ11* gene product was found to regulate insulin secretion; *TCF7L2* is involved in survival and functioning of beta cells (Shu et al. 2008) and insulin secretion, while *GRL* plays role in beta cell function. Polymorphisms in these genes have been intensively studied in individuals of different ethnic origins. Results show that genetic variants of *TCF7L2* and *KCNJ11* genes have potential to emerge as a risk biomarker for T2DM, whereas results of *GNB3*, *GRL* and *NET* genes have been controversial when studied in individuals of different ethnicities.

**Table 1:** Significance of *GNB3*, *NET*, *KCNJ11*, *TCF7L2* and *GRL* genes polymorphism with T2DM in various ethnic groups

S. no.	Gene	Polymorphism	Ethnicity	Case/control (*n*)	Significance	References
1	*GNB*	*C825T (rs 5443)*	Arab	256/254	Y	Kiani et al. (2005)
		*C825T (rs 5443)*	Caucasian	1358/4723	Y	Andersen et al. (2006)
		*C825T (rs 5443)*	Japanese	472/388	Y	Hayakawa et al. (2007)
		*C825T (rs 5443)*	Japanese	230/2576	Y	Daimon et al. (2008)
		*C825T (rs 5443)*	Asian Indian	576	Y	Pemberton et al. (2008)
		*C825T (rs 5443)*	Hispanic American population	185/261	N	Parra et al. (2004)
		*C825T (rs 5443)*	Danish	4387/3131	N	Andersen et al. (2006)
		*C825T (rs 5443)*	South Indians	70/70	N	Chandrasekaran et al. (2012)
		*C825T (rs 5443)*	Canadian	522	Y	Pollex et al. (2006)
		*C825T (rs 5443)*	Greek	226/110	N	Maniotis et al. (2014)
2	*NET*	*G1287*	Caucasians	310/360	N	Ksiazek et al. (2006)
3	*KCNJ11*	*E23K (rs5219)*	Chinese Han population	502/252	Y	Liu et al. (2006)
		*rs5210*	Chinese Han population	502/252	Y	Liu et al. (2006)
		*rs5210*	Korean	761/630	Y	Koo et al. (2007)
		*rs5215*	Chinese Han population	502/252	Y	Liu et al. (2006)
		*rs5215*	North Indians	1019/1006	Y	Chavali et al. (2011)
		*rs5215*	Japanese	909/893	Y	Sakamoto et al. (2007)
		*rs5219*	Mauritanian	135/135	Y	Abdelhamid et al. (2014)
			Chinese and East Asian	1912/2041	Y	Zhou et al. (2009)
		*rs886288*	Korean	761/630	Y	Koo et al. (2007)
		*rs12255372* and *rs7903146*	Finnish	1151/953	Y	Scott et al. (2006)
		*rs7901695 rs7903146*	Amish	276/342	Y	Damcott et al. (2006)
		*rs7901695* and *rs7903146*	Amish	276/342	Marginal association	Damcott et al. (2006)
		*rs12255372*	U. S	687/1051	Y	Zhang et al. (2006)
		*rs11196205*	Chinese	433/419	Y	Ng et al. (2007)
		*rs7903146*	Brazilian	190/370	Y	Marquezine et al. (2008)
		*rs12255372 rs12255372 rs7903146 rs7901695*	Japanese	1630/1064	Y	Hayashi et al. (2007)
		*rs12255372 rs7903146*	Dutch	502/920	Y	van Vliet-Ostaptchouk et al. (2007)
		*rs7903146*	Iranian	258/168	Y	Amoli et al. (2010)
		*rs12255372 rs4506565 rs7903146*	Indians	955/399	Y	Chandak et al. (2007)
4	*TCF7L2*	rs7903146	Danish	31/31	Y	Gjesing et al. 2011
		*rs12255372*	Europeans Caucasians Asians	34,076/36,192	Y	Wang et al. (2013)
			Africans		N	
		*rs12255372*	Cameroonian	60/60	Y	Nanfa et al. (2015)
		*rs7903146*	Moroccans	504/406	Y	Cauchi et al. (2007)
		*rs7903146*	Austrians	486/1075	Y	
		*rs7903146 rs12255372*	French	2367/2499	Y	Cauchi et al. (2006)
		*rs7903146 rs12255372 rs11196205*	South Indian	758/621	Y	Jyothi et al. (2013)
		*rs7901695, rs7903146, rs7895340, rs11196205, rs12255372*	African Americans	577/632	Y	Sale et al. (2007)
		*rs12255372 rs7903146*	Finnish	1151/953	Y	Scott et al. (2006)
		*rs11196218*	Asian	1842/4444	Y	Zhai et al. (2014)
		*rs7903146 rs11196205 rs12255372*	Japanese	2214/1873	Y	Miyake et al. (2008)
		*rs290487*	Han Chinese	760/760	Y	Chang et al. (2007)
		*rs7903146 rs12255372*	Han Chinese	760/760	N	Chang et al. (2007)
		*rs7903146 rs7901695 rs12255372*	Italian	154/171	Y	Cincia et al. (2012)
		*rs12255372 rs7903146*	Arabs	522/346	N	Alsmadi et al. (2008a)
		*rs7903146 rs12255372 rs290487*	Kurdish	173/173	Y	Shokouhi et al. (2014)
5	*GRL*	*A1220G; N363S*	French Caucasians	369	N	Roussel et al. 2003
		*Bcl I*	Swedish men	163	Y	Rosmond and Holm 2008
		*Tth111I*	Swedish men	163	N	
		*A/G*	Swedish men	163	N	
		*Bcl I*	British Caucasian	56/43	Y	Weaver et al. (1992)
		*A3669G*	Italians	61/71	Y	Trementino et al. (2012)
		*BclI, N363S ER22/23EK*	Italians	61/71	N	Trementino et al. (2012)

### Guanine nucleotide-binding protein subunit beta 3 (GNB3) gene

G proteins also known as heterotrimeric guanine nucleotide-binding proteins are signaling molecules which are involved in regulating blood glucose levels through metabolic pathway of insulin signaling. They are also involved in the stimulation of secondary messengers such as adenylate cyclase, signaling pathways of epinephrine and glucagon receptors in hepatic, muscle and fat-tissue cells which are the pathophysiological signaling pathways leading to T2DM. These heterotrimeric protein molecules are made up of 3 subunits, alpha (α), beta (β) and gamma (γ) (Hurowitz et al. 2000). These G protein-coupled receptors activate a downstream secondary messenger cascade on binding ligand molecules such as hormones, neurotransmitters, chemokines, local mediators, etc. (Chandrasekaran et al. 2012). The *GNB3* gene codes for the beta3 subunit of G proteins. Any change in the *GNB3* gene due to any mutation or polymorphism may possibly lead to defects in the G protein encoded by the defective gene. These polymorphic changes have been found to be associated with the development of T2DM as well as its associated secondary complications. In this context, the most studied polymorphism of *GNB3* gene is the C825T polymorphism. The 825T variant of *GNB3* gene occurs when cytosine is replaced with thymine at position 825 in exon 10 in the wild-type gene due to alternative splicing leading to loss of 41 amino acids. The mutant 825T allele was associated with an increased production of Gβ3s (Andersen et al. 2006), resulting in enhanced signal transduction by G proteins. Studies found that the *GNB3* C825T variant is a risk factor for hypertension, obesity, metabolic syndrome, atherosclerosis and diabetes (Siffert 2005). Various studies have confirmed the association of *GNB3* polymorphism with T2DM in different populations. In Japanese population, multiple logistic regression analysis showed a significant association of the genotypes TT + TC with T2DM (Daimon et al. 2008). However, the results have been shown to be controversial in this population where another study on the same population found no significant association (Hayakawa et al. 2007). A study on Emirati and Canadian population found that 825T variant showed a positive association with T2DM risk (Kiani et al. 2005; Pollex et al. 2006). Numerous studies have showed a strong association of *GNB3* polymorphism with the risk of diabetic neuropathy in different ethnic groups (Beige et al. 2000; Blüthner et al. 1999; Zychma et al. 2000). In contrast, some studies have also showed no association of *GNB3* polymorphism with T2DM in Hispanic American (Parra et al. 2004), Danish (Andersen et al. 2006), Greek (Maniotis et al. 2014) and South Indian Populations (Chandrasekaran et al. 2012).

### Norepinephrine transporter (NET) gene

The norepinephrine transporter (NET), also known as solute carrier family 6 member 2 (SLC6A2) (Pacholczyk et al. 1991). It is a 617 amino acid multipass monoamine symporter protein encoded by *SLC6A2* gene present on chromosome 16 (Gelernter et al. 1993). It is involved in sodium–chloride dependent active reuptake of norepinephrine, a potent neurotransmitter, at neuronal junctions into the presynaptic nerve terminals. It helps in maintaining norepinephrine homeostasis in our body by regulating its metabolism and turnover rates. A potential decrease in norepinephrine uptake sites due to dysfunctional transporter had been observed in various cardiovascular diseases, such as hypertension, cardiomyopathy as well as in diabetes mellitus (Hahn and Blakely 2002). This could possibly occur as a result of various mutations and polymorphisms where a single nucleotide changes in the gene neither encoding nor epinephrine transporter may result in a non functional protein. A lot of studies have been conducted globally to find a potential relationship between single nucleotide polymorphism in *NET* gene and various diseases such as psychiatric disorders (Tellioglu and Robertson 2001), hyperactivity disorder (Kim et al. 2006), ADHD (Shannon et al. 2000; Neubauer and Christensen 1976) orthostatic intolerance (Tellioglu and Robertson 2001; Shannon et al. 2000) etc. Neubauer in 1976 first reported that norepinephrine concentration reduces considerably in the cardiovascular system of the diabetic patients but till date an insignificant amount of data is present to provide a potential evidence of the association of *NET* gene polymorphism with T2DM. The first ever study in this field was first carried out in 2006 on the G1287 single nucleotide polymorphism present in exon 9 of *NET* gene and T2DM in Caucasians where no significant association was found between this gene polymorphism and the risk of the development of T2DM and diabetic nephropathy (Ksiazek et al. 2006). However, it was observed that the AA genotype might contribute to the development of hypertension in these diabetic subjects. The frequency of AA genotype is reported to be higher in diabetic patients suffering from hypertension when compared with normotensive diabetic patients (19 vs. 10%, respectively, *p* < 0.05, OR = 2.21 with 95% CI 1.36–3.59) (Ksiazek et al. 2006). To the best of my knowledge this is the only study conducted so far between *NET* gene polymorphism and T2DM which calls for further association studies and testing of coding region variants of this gene and their functional assessment.

### Potassium channel (KCNJ11) gene

The Potassium Channel gene also known as the Potassium inwardly rectifying channel, subfamily J, member 11 potassium channel gene and abbreviated as *KCNJ11* belongs to potassium channel gene family. It is located at 11p15.1 on human chromosome and don’t have any intronic sequences (Haghvirdizadeh et al. 2015). This gene encodes a 390 amino acid protein which is an inward-rectifier potassium ion channel called Kir6.2. The Kir6.2 protein along with another protein SUR1 encoded by the *ABCC8* gene located next to the *KCNJ11* gene forms the KATP channel which modulates insulin production and secretion through glucose metabolism (McTaggart et al. 2010) and is the target of sulphonylurea drugs used for treating diabetes. Genetic polymorphism in *KCNJ11* gene has been shown to be associated with an increased risk of T2DM. The *KCNJ11* gene variant (Glu23Lys or E23K) where glutamic acid is replaced with lysine at 23rd position in the translated protein had been found to be a potential biomarker for T2DM (Qiu et al. 2014). This variant replaces the amino acid glutamic acid with the amino acid lysine at position 23 in the protein sequence, written as Glu23Lys or E23K. The lysine carrier having the variant gene were found to have reduced insulin secretion but were less likely to develop T2DM (Florez et al. 2007). A case control and a meta-analysis on Tunisian and Arab population case-also revealed significant association between *KCNJ11* E23K variant and T2DM (Lasram et al. 2014). Recent study on Iranian subjects demonstrated that the carriers homozygous for KK genotype of *KCNJ11* gene were susceptible to T2DM (*p* value = 0.049) and the frequency of K allele was higher in T2DM patients than control subjects (Rastegari et al. 2015). Another study on arab population showed significant association of the KCNJ11 E23K polymorphism with type 2 diabetes (Alsmadi et al. 2008a). A meta-analysis which included 48 published studies found a higher disease risk among Caucasians and East Asians carrying the lysine variant but no significant association was found with Indian and other ethnic populations (Qiu et al. 2014). Studies on Russian and Mauritanian populations also found an association between E23K variant and T2DM (Nikitin et al. 2015; Abdelhamid et al. 2014). However, apart from above mentioned studies a contrasting finding was observed in Iranian population where *KCNJ11* E23K polymorphism is not associated with genetic susceptibility to T2DM but it was inferred that it might play a role in the progression of T2DM in obese subjects (Keshavarz et al. 2014). Similarly no significant association was observed in Moroccan population (Benrahma et al. 2014). Also, it was shown that patients carrying the lysine variant were more prone to secondary failure to sulphonylurea, T2DM treatment drug leading to higher fasting plasma glucose and glycosylated hemoglobin concentrations (Sesti et al. 2006).

### Transcription factor 7-like 2 (TCF7L2) gene

Transcription factor 7-like 2 (*TCF7L2*) is a 215.9 kb gene located at 10q25 on human chromosome (Duval et al. 2000). TCF7L2 is a major component of Wnt signaling pathway, which is involved in various cellular growth and developmental processes. TCF7L2 regulates various pathways such as adipogenesis, development of pancreatic islet cells, survival and functioning of beta cells (Shu et al. 2008). It also plays a major role in insulin secretion by regulating various proteins involved in postprandial insulin secretion such as proglucagon and glucagon-like peptides GLP-1 and GLP-2 at transcriptional level (da Silva Xavier et al. 2009; Doria et al. 2008). *TCF7L2* variants are regarded as the strongest genetic risk factors for T2DM, since it increase the disease risk 1.5-fold in heterozygotes and 2.4-fold in homozygotes, contributing to a 21% population risk (Grant et al. 2006). However, the *TCF7L2* disease risk variants were found to vary among different populations (Guinan 2012). The most studied polymorphisms in *TCF7L2* gene are rs7903146 of the intron 3 (IVS3C > T), rs7901695 of the intron 3 (IVS3T > C), rs12255372 of the intron 4 (IVS4G > T) and rs11196205 of the intron 4 (IVS4G > C). Polymorphism in *TCF7L2* gene disrupts body glucose homeostasis by impairing insulin secretion, glucose turnover and glucose tolerance pathways by directly affecting pancreatic beta cells (Lyssenko et al. 2007; Schafer et al. 2007). In another study genetic polymorphism in *TCF7L2* gene (rs7903146) was shown to predispose an individual to the risk of developing T2DM where the presence of TT genotype results in elevated plasma glucose, serum proinsulin and plasma gastric inhibitory polypeptide levels (Gjesing et al. 2011). *TCF7L2* variants have been found to have a positive association with T2DM in French (Cauchi et al. 2006), African-American (Sale et al. 2007), Finnish (Scott et al. 2006), U. K. (Groves et al. 2006), Japanese (Miyake et al. 2008), Indian (Chandak et al. 2007) and Chinese (Chang et al. 2007) populations. Three *TCF7L2* polymorphisms rs7903146, rs7901695 and rs12255372 were found to be associated with T2DM in Italian population and a strong correlation was found between rs7903146 and cardiovascular autonomic neuropathy in the study subjects (Cincia et al. 2012). In Kurdish population of Iran the T-allele of rs12255372, rs7903146, and rs290487 polymorphisms of *TCF7L2* were found to be a risk allele for T2DM (Shokouhi et al. 2014). A large meta-analysis demonstrated significant association between IVS3C > T as well as IVS4G > T *TCF7L2* gene polymorphisms and T2DM where among IVS3C > T polymorphism, TC heterozygotes and TT homozygous variants increased risk of T2MD by 1.4-fold and 2.0-fold, respectively, as compared to wild-type CC homozygotes whereas in case of IVS4G > T polymorphism, TG heterozygotes and TT homozygous variants increased risk of T2MD by 1.4-fold and 1.9-fold, respectively, as compared to wild-type GG homozygotes (Tong et al. 2009). In contrast, no significant association was found between *TCF7L2* variants and T2DM among East Asians and conflicting results have appeared, in various studies on Chinese population (Chang et al. 2007; Ng et al. 2007). In Arabs, weak or no association was observed in two polymorphisms of *TCF7L2* gene (rs12255372, rs7903146) with T2DM (Alsmadi et al. 2008b).

### Glucocorticoid receptor (GRL) gene

The glucocorticoid receptor gene (*GRL*) also known as *NR3C1* (nuclear receptor subfamily 3, group C, member 1) gene, is located on chromosome number 5 at 5q31 position in humans. (Marti et al. 2006) It encodes a 777-amino acid (Glucocorticoid receptor α) or a 742-amino acid (Glucocorticoid receptor β) polypeptide which acts as a receptor for cortisol and glucocorticoids and regulates gene expression of various genes involved in cell development, metabolism and immune responses. The 363S allele of the *N363S* variant present in exon 2 of *GRL* gene was found to be associated with the risk of weight gain/obesity but no evidence was found for an association of this variant with parameters related to hyperglycaemia in French Caucasians with T2DM (Roussel et al. 2003). In another study on Swedish men, significant increase in fasting glucose and insulin were found over the 5-year follow-up among individuals homozygotes for the BclI allele of *GRL* gene whereas no significant associations were found with Tth111I or A/G polymorphism of *GRL* gene with T2DM (Rosmond and Holm 2008). Similar results were reported in Italian subjects with Addison’s disease where patients carrying GG genotype of BclI polymorphism showed higher glucose levels or glucose intolerance as compared to individuals carrying wild-type CC and heterozygous CG genotype (Giordano et al. 2012). Another study on British Caucasian obese women showed that Bc1I polymorphism was significantly associated with higher fasting insulin and index of insulin resistance (HOMA) (weaver et al. 1992). In a different study on Italian patients with Cushing’s syndrome the A3669G polymorphism of *GRL* gene was seen to have a protective role where it was found to decrease the risk of developing T2DM (Trementino et al. 2012). In a cross-sectional cohort study on N363S (rs6195), BclI (rs41423247), ER22/23EK (rs6189/6190), 9b A/G (rs6198) and ThtIIII (rs10052957) polymorphisms in KCNJ11 gene, none of the polymorphisms studied was found to be associated with insulin sensitivity, however, *N363S* and *ER22/23EK* polymorphisms of the *GRL* gene were found to be negatively associated with β-cell function in women, but not in men (van Raalte et al. 2012).

## Conclusion

The incidence of diabetes occurrence is increasing at a fast pace globally and present data demonstrate that its prevalence is also increasing among children and adolescents who are below 30 years of age. This calls for an urgent development of techniques and methods to combat this growing epidemic and to identify high-risk individuals at an early stage that could thus be prevented from T2DM by taking suitable preventive measures. Apart from environmental factors such as diet, ethnicity, family history, lifestyle, etc., genetic factors also have a profound effect on the occurrence of T2DM and they interact with the environmental factors in predisposing an individual to T2DM. Thus, a susceptible gene in one population might not show the same phenotypic effect in other population. Various genetic linkage and associations studies have identified several candidate genes for T2DM and ongoing studies on in this field are focusing on identifying novel gene variants which are having a potential role in the pathophysiology of T2DM. This review had summarized the results from various global studies on the association of *GNB3*, *NET*, *KCNJ11*, *TCF7L2* and *GRL* genes with T2DM which could possibly help researchers working in this field and would eventually help in understanding the mechanistic pathways of T2DM. Results show that genetic variants of *TCF7L2* and *KCNJ11* genes have potential to emerge as a risk biomarker for T2DM whereas polymorphisms in *GNB3*, *GRL* and *NET* genes may confer smaller or modifier effects since the results have been controversial and will require further larger studies to fully elucidate their role in T2DM. Thus, we could say that a proper understanding of genetic background of T2DM will help in understanding the biochemical and molecular mechanisms, developing potential biomarkers which could identify at-risk patients in early stages and designing new therapeutics which will thus help in diagnostics, treatment and eventually prevention of this disease.
